# Berberine Ameliorates Prenatal Dihydrotestosterone Exposure-Induced Autism-Like Behavior by Suppression of Androgen Receptor

**DOI:** 10.3389/fncel.2020.00087

**Published:** 2020-04-09

**Authors:** Dongfang Xiang, Jianping Lu, Chongxia Wei, Xiaofan Cai, Yongxia Wang, Yujie Liang, Mingtao Xu, Zichen Wang, Min Liu, Min Wang, Xuefang Liang, Ling Li, Paul Yao

**Affiliations:** ^1^The Second Affiliated Hospital of Guangzhou University of Chinese Medicine, Guangzhou, China; ^2^Department of Child Psychiatry, Kangning Hospital of Shenzhen, Shenzhen, China; ^3^Hainan Maternal and Child Health Hospital, Haikou, China

**Keywords:** autism spectrum disorders, berberine, androgen, PCOS, autism-like behavior

## Abstract

Many epidemiology studies have shown that maternal polycystic ovary syndrome (PCOS) results in a greater risk of autism spectrum disorders (ASD) development, although the detailed mechanism remains unclear. In this study, we aimed to investigate the potential mechanism and provide a possible treatment for PCOS-mediated ASD through three experiments: Experiment 1: real-time PCR and western blots were employed to measure gene expression in human neurons, and the luciferase reporter assay and chromatin immunoprecipitation (ChIP) was used to map the responsive elements on related gene promoters. Experiment 2: pregnant dams were prenatally exposed to dihydrotestosterone (DHT), androgen receptor (AR) knockdown (shAR) in the amygdala, or berberine (BBR), and the subsequent male offspring were used for autism-like behavior (ALB) assay followed by biomedical analysis, including gene expression, oxidative stress, and mitochondrial function. Experiment 3: the male offspring from prenatal DHT exposed dams were postnatally treated by either shAR or BBR, and the offspring were used for ALB assay followed by biomedical analysis. Our findings showed that DHT treatment suppresses the expression of estrogen receptor β (ERβ) and superoxide dismutase 2 (SOD2) through AR-mediated hypermethylation on the ERβ promoter, and BBR treatment suppresses AR expression through hypermethylation on the AR promoter. Prenatal DHT treatment induces ERβ suppression, oxidative stress and mitochondria dysfunction in the amygdala with subsequent ALB behavior in male offspring, and AR knockdown partly diminishes this effect. Furthermore, both prenatal and postnatal treatment of BBR partly restores prenatal DHT exposure-mediated ALB. In conclusion, DHT suppresses ERβ expression through the AR signaling pathway by hypermethylation on the ERβ promoter, and BBR restores this effect through AR suppression. Prenatal DHT exposure induces ALB in offspring through AR-mediated ERβ suppression, and both prenatal and postnatal treatment of BBR ameliorates this effect. We conclude that BBR ameliorates prenatal DHT exposure-induced ALB through AR suppression, this study may help elucidate the potential mechanism and identify a potential treatment through using BBR for PCOS-mediated ASD.

## Background

Autism spectrum disorders (ASD) are a group of neurodevelopmental disorders characterized by impaired social communication and interaction, together with restrictive and repetitive behaviors (Rossignol and Frye, 2012; Bralten et al., 2018; Hoirisch-Clapauch and Nardi, 2019). It has been reported that prenatal androgen levels are associated with ASD, and fetal testosterone predicts sexually-differentiated childhood behavior (Tordjman et al., 1997; Auyeung et al., 2009; Ruta et al., 2011). Furthermore, our recent studies have shown that suppression of estrogen receptor β (ERβ) and its target gene is associated with ASD development, while the expression of estrogen receptor α (ERα) has little correlation (Zou et al., 2017; Li et al., 2018; Xie et al., 2018), leading us to suppose that prenatal androgen exposure may induce ASD development through ERβ suppression.

Polycystic ovary syndrome (PCOS) is a common endocrine disorder in women characterized by hyperandrogenism and ovarian dysfunction (Kosidou et al., 2017). Many epidemiology studies have shown that maternal PCOS is associated with a greater risk of ASD (Kosidou et al., 2016; Cherskov et al., 2018; Katsigianni et al., 2019); furthermore, prenatal androgen exposure may affect the neurodevelopment of offspring, resulting in ASD and attention-deficit/hyperactivity disorder (ADHD; Baron-Cohen et al., 2011; Berni et al., 2018; Cesta et al., 2020), while the detailed mechanism for PCOS-mediated ASD remains unclear.

Recently, the rodent PCOS model with chronic androgen treatment displays many reproductive and metabolic characteristics that are similar to that of human PCOS and provides many advantages for PCOS research, including its inexpensive cost, stable genetic background, shorter life span and generation time, et cetera (Ruta et al., 2011; Walters et al., 2012; Noroozzadeh et al., 2017; Iwasa et al., 2020). Currently, three androgen-based PCOS models are typically used, including testosterone (T), dihydrotestosterone (DHT) and dehydroepiandrosterone (DHEA), and each of these models has its strengths and weaknesses (Walters et al., 2012), which we considered in the selection of the androgen for our model. DHT is a non-aromatizable androgen that cannot be converted into estradiol so that there is no interference from estrogen (Noroozzadeh et al., 2017). In this study, we chose the DHT-induced rat as the PCOS research model.

Berberine (BBR) is a small isoquinoline alkaloid molecule that can be isolated from plants and holds many pharmacological activities, including antimicrobial, antitumoral, and metabolic modulation effects (Cai et al., 2016). It passes through the blood-brain barrier (BBB) and has many positive effects against some neurodegenerative diseases, including Alzheimer’s disease (AD) and Parkinson’s disease (PD; Jiang et al., 2015; Cai et al., 2016). Also, BBR has been reported to suppress the androgen receptor (AR) signaling pathway (Li et al., 2011) and prostatic hyperplasia (Youn et al., 2018), and has many potential effects on PCOS (Li et al., 2013; An et al., 2014; Arentz et al., 2014; Wang et al., 2016), although the detailed mechanism remains unclear and there have been no reports on the effect of BBR on ASD development.

Here, we aim to investigate the mechanism and effect of BBR on PCOS-mediated ASD. The *in vitro* cell culture study shows that DHT (Berkel et al., 2018) suppresses expression of ERβ and its target gene superoxide dismutase 2 (SOD2; Liu et al., 2014) in human neurons through AR expression, and BBR restores this effect through AR suppression. Further investigation shows that DHT/BBR-mediated gene suppression is due to hypermethylation on the related gene promoters. We further established an *in vivo* PCOS rat model by DHT administration (Mannerås et al., 2007; Paixão et al., 2017) and found that maternal PCOS induces autism-like behavior (ALB) in offspring through ERβ/SOD2 suppression and subsequent oxidative stress and mitochondrial dysfunction. Prenatal treatment of AR knockdown restores this effect, indicating that prenatal DHT exposure induces ALB through the AR signaling pathway; interestingly, both prenatal and postnatal treatment of BBR partly restores this effect. We conclude that BBR ameliorates prenatal DHT exposure-induced ALB by AR suppression. Although it is important to note that the DHT-induced rat model may not completely mimic the PCOS situation in humans, these findings may indicate a potential mechanism and treatment for PCOS-mediated ASD, paving the way for a new avenue of research in this field.

## Materials and Methods

A detailed description can be found in Supplementarty Data S1, and the primers used in this study are shown in Supplementary Table S1. The human neural progenitor cell ACS-5003 was obtained from ATCC and cultured in NPC medium with supplements and antibiotics. In some experiments, the cells were conditionally immortalized using an hTERT lentivirus vector with an extended life span to achieve higher transfection efficiency and experimental stability (Bodnar et al., 1998; Kong et al., 2016).

### Construction of Human AR/ERβ Reporter Plasmid

To construct AR/ERβ reporter plasmids, the AR/ERβ gene promoters (2 kb upstream of the transcription start site plus first exon) were amplified from Ensembl gene ID: ARLNC1-205 ENST00000650661.1 (for AR) and ESR2-201 ENST00000267525.10 (for ERβ) by PCR from human genomic DNA and subcloned into the pGL3-basic vector (#E1751, Promega) using restriction sites of Mlu I and Hind III with the following primers: AR forward: 5′-gcgc-acgcgt- cga ggt tag gag ata aag acc -3′ (Mlu I) and AR reverse: 5′- gtac- aagctt- ctt ttc tgt aca tct cta gat -3′ (Hind III); ERβ forward: 5′-gcgc-acgcgt- atttcaagacgagcctggcca -3′ (Mlu I) and ERβ reverse: 5′- gtac- aagctt- ctg ttt aca ggt aag gtg tgt -3′ (Hind III). To map AR or ERβ promoter activity, the related deletion promoter constructs were generated by PCR methods and subcloned into the pGL3-basic vector (Zhang et al., 2017).

### Preparation of Human AR Expression Lentivirus

The cDNA for human AR (obtained from Open Biosystems) was subcloned into the pLVX-Puro vector (from Clontech) with the restriction sites of Xho1 and Xba1 using the below primers: human AR forward primer: 5′- gtac-ctcgag- atg gaa gtg cag tta ggg ctg -3′ (Xho1) and human AR reverse primer: 5′- gtac-tctaga-tca ctg ggt gtg gaa ata gat -3′ (Xba1). The lentivirus for either AR or empty control (CTL) was expressed by Lenti-X™ Lentiviral Expression Systems (from Clontech).

### Preparation of shAR Knockdown Lentivirus

The shRNA lentivirus plasmid for human AR (sc-29204-SH), or non-target control (sc-108060) were purchased from Santa Cruz Biotechnology, and the shRNA lentivirus plasmid for rat AR was a kind gift from Dr. Haimou Zhang (from Hubei University). The related lentivirus for shAR or empty control (CTL) were expressed through Lenti-X™ Lentiviral Expression Systems (from Clontech) per manufacturers’ instructions. The purified and condensed lentivirus was used for *in vivo* gene knockdown. The knockdown efficiency was confirmed by more than 65% of mRNA reduction compared to the control group in rat amygdala cells using real-time PCR (see Supplementary Table S1).

### Luciferase Reporter Assay

1.0 × 10^5^ of treated cells were seeded in a 6-well plate with complete medium to grow until they reached 80% confluence. Cells were then cotransfected by 3 μg of reporter constructs, together with 0.2 μg of pRL-CMV-Luc *Renilla* plasmid (from Promega). After treatment, the cells were harvested and the luciferase activity assays were carried out using the Dual-Luciferase™ Assay System (Promega), and the transfection efficiencies were normalized using a cotransfected *Renilla* plasmid according to manufacturers’ instructions. The reporter activities for AR and ERβ were calculated (Zhang et al., 2017).

### Chromatin Immunoprecipitation (ChIP)

Cells were washed and cross-linked using 1% formaldehyde for 20 min and terminated by 0.1 M glycine. Cell lysates were sonicated and centrifuged. Five-hundred micrograms of protein were pre-cleared by BSA/salmon sperm DNA with preimmune IgG and a slurry of Protein A Agarose beads. Immunoprecipitations were performed with the indicated antibodies, BSA/salmon sperm DNA and a 50% slurry of Protein A agarose beads. Input and immunoprecipitates were washed and eluted, then incubated with 0.2 mg/ml Proteinase K for 2 h at 42°C, followed by 6 h at 65°C to reverse the formaldehyde crosslinking. DNA fragments were recovered through phenol/chloroform extraction and ethanol precipitation. A −150 bp fragment in the range of −300~0 from the transcription start site on the AR/ERβ promoter was amplified by quantitative real-time PCR (qPCR) using the primers provided in Supplementary Table S1 (Zhang et al., 2017; Zou et al., 2017).

### *In vivo* Rat Experiments

Sprague–Dawley rats were obtained from Guangdong Medical Animal Center, and maintained under standard 12 h light/dark cycles and given *ad libitum* access to food and water.

#### Rat Protocol 1: Prenatal Treatment

The 2-month female rats were anesthetized and received treatments consisting of 60-day release pellets that were implanted on the dorsal neck. Hormone pellets contained 5 mg of either (DHT, #A-161), or vehicle pellets (CTL) containing the same matrix but with no hormone (Moran et al., 2007). Furthermore, the rats were implanted with a guide cannula targeting the amygdala (26 gauge; Plastics One; Neal-Perry et al., 2014; Hu et al., 2015). An osmotic minipump (Alzet model 2002; flow rate 0.5 μl/h; Cupertino, CA, USA) connected to a 26-gauge internal cannula that extended 1 mm below the guide was implanted and used to deliver AR knockdown (shAR) or empty (EMP) lentivirus. A vehicle consisting of artificial cerebrospinal fluid (aCSF) was used for the infusion of the lentivirus. Infusion (flow rate 0.5 μl/h) begun immediately after the placement of the minipump. 0.5 μl of total 2 × 10^3^ cfu of lentivirus was infused for 1 h. After 1 week of surgery recovery, the female rats were mated with proven male rats, and the successful pregnancy was confirmed by examining the vaginal plugs. The dams were then treated with vehicle (DMSO, 1 ml/kg) or BBR (10 mg/kg, dissolved in DMSO) by intraperitoneal (i.p.) injection every 2 days starting from day 1. The experimental rats were separated into 4 groups (10 per group). Group 1: CTL rats with empty control lentivirus infusion (CTL); Group 2: DHT rats with empty control lentivirus infusion (DHT); Group 3: DHT rats with shAR knockdown lentivirus infusion (DHT/shAR); Group 4: DHT rats with BBR injection (DHT/BBR). The amygdala neurons were isolated on embryonic day 18 (E18) as described below. After birth, the male offspring were separated from the dams on day 21 and fed until 7–8 weeks old for further experiments. The offspring were then used for ALB tests followed with biomedical analysis (Zou et al., 2017).

#### Rat Protocol 2: Postnatal Treatment

The 2-month female rats were prenatally exposed to either CTL or DHT during their 21-day pregnancy period as described by Rat Protocol 1. Two-week old male offspring from the above dams were infused with either AR knockdown (shAR) or empty (EMP) lentivirus or treated with vehicle (VEH, 1 ml/kg of DMSO) or BBR (10 mg/kg, dissolved in DMSO) by intraperitoneal (i.p.) injection every 2 days as mentioned in Rat Protocol 1. The rats were separated into the below four groups (10 per group). Group 1: CTL rats with empty control lentivirus infusion (CTL); Group 2: DHT rats with empty control lentivirus infusion (DHT); Group 3: DHT rats with postnatal shAR knockdown lentivirus infusion (DHT/p-shAR); Group 4: DHT rats with postnatal BBR injection (DHT/p-BBR). After 2 weeks of lentivirus and BBR treatment, the offspring were then used for behavior tests and biomedical analysis, as discussed in Rat Protocol 1 (Zou et al., 2017).

### Animal Behavior Test

The animal behavior test of offspring was carried out at 7–8 weeks of age. ALB was evaluated using ultrasonic vocalizations (Silverman et al., 2010; Schaafsma et al., 2017), social recognition tests (Moy et al., 2004; Schaafsma et al., 2017) and a three-chambered social test (Moy et al., 2004; Silverman et al., 2010; Schaafsma et al., 2017; Choi et al., 2019; Wang et al., 2019).

### Immunostaining

The isolated amygdala neurons were transferred to coverslips for incubation under growth conditions before being washed with PBS, fixed in 4% paraformaldehyde for 20 min, and incubated with 0.3% Triton X-100 in PBS for 15 min. After blocking with normal goat serum, sections with 8-oxo-dG anti-mouse antibody (1:100, #4354-MC-050, from Novus Biologicals) were incubated for 12 h at 4°C and subsequently with secondary antibody Alexa Fluor 488. The coverslips were then mounted by antifade Mountant with DAPI (staining nuclei, in blue). The photographs were taken using a Confocal Laser Microscope (Leica, 20× lens) and quantitated using ImageJ software.

## Results

### BBR Restores DHT-Mediated ERβ/SOD2 Suppression by AR Suppression in Human Neurons

**Figure 1:**
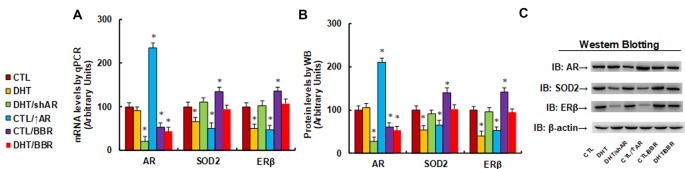
Berberine (BBR) restores dihydrotestosterone (DHT)-mediated estrogen receptor β/superoxide dismutase 2 (ERb/SOD2)/SOD2 suppression by androgen receptor (AR) suppression in human neurons. The human ACS-5003 neurons were treated by control (CTL), DHT (10 nM), DHT with AR knockdown by lentivirus (DHT/shAR), CTL with AR overexpression by lentivirus (CTL/↑AR), CTL with 10 μM BBR (CTL/BBR), or DHT with BBR (DHT/BBR) for 24 h, then the cells were harvested for further analysis. **(A)** mRNA levels for AR, ERβ and SOD2 by qPCR, *n* = 4. **(B)** Quantitation of protein levels, *n* = 5. **(C)** Representative western blotting pictures for **(B)**. *P < 0.05, vs. CTL group. Data were expressed as mean ± SEM.

We evaluated the potential effect of DHT and BBR on the gene expression of AR, SOD2, and ERβ. The human ACS-5003 neurons were treated by either control (CTL), DHT (10 nM), or DHT with AR knockdown by lentivirus (DHT/shAR), or CTL with AR overexpression by lentivirus (CTL/↑AR), CTL with 10 μM BBR (CTL/BBR), or DHT with BBR (DHT/BBR) for 24 h, then cells were harvested for gene expression analysis. Our results showed that DHT treatment decreased expression of SOD2 and ERβ to 66% and 51%, respectively compared to the CTL group, while it did not have any effect on AR expression. We then evaluated the effect of shAR on DHT treatment (DHT/shAR). Our results showed that shAR decreased AR expression to 21%, indicating a successful AR gene manipulation by lentivirus knockdown; very interestingly, shAR completely restored DHT-mediated gene suppression of SOD2 and ERβ. We also measured the effect of AR overexpression (CTL/↑AR) and found that CTL/↑AR increased AR expression to 235% compared to CTL group, indicating a successful gene manipulation by lentivirus overexpression; furthermore, it decreased expression of SOD2 and ERβ to 51% and 47%, respectively. We finally measured the effect of BBR (CTL/BBR), and the results showed that CTL/BBR and DHT/BBR decreased AR expression to 53% and 44%, respectively, and CTL/BBR increased expression of SOD2 and ERβ to 135% and 136%, respectively, compared to the CTL group. Our results indicate that DHT suppresses the expression of SOD2 and ERβ, and AR knockdown restores, while AR expression mimics, this effect; BBR restores this effect by AR suppression (see Figure 1A). We then measured the protein levels for these genes, and an expression pattern similar to that of the mRNA levels was observed (see Figures 1B,C, Supplementary Figure S1A). We conclude that BBR restores DHT-mediated ERβ/SOD2 suppression by AR suppression.

### DHT-Mediated ERβ Suppression Is Due to DHT-Induced Histone Methylation on the ERβ Promoter

**Figure 2:**
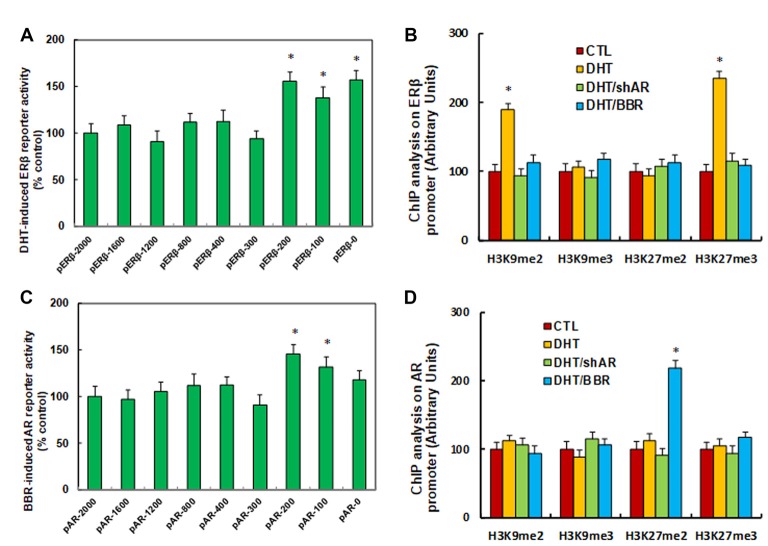
DHT-mediated ERβ suppression and BBR-mediated AR suppression by histone methylation on the promoters. **(A)** The conditional immortalized ACS-5003 neurons were transiently transfected by either ERβ full length (pERβ-2000) or deletion reporter plasmids. After 24 h, the cells were treated by either control (CTL) or 10 nM DHT for 24 h, and DHT-induced relative reporter activities (% control) were calculated, *n* = 4. *P < 0.05, vs. pERβ-2000 group. **(B)** The human ACS-5003 neurons were treated by CTL, 10 nM DHT (DHT), DHT with AR knockdown by lentivirus (DHT/shAR), or DHT with BBR (DHT/BBR) for 24 h, and cells were harvested for ChIP analysis, *n* = 4. *P < 0.05, vs. CTL group. **(C)** The conditional immortalized ACS-5003 neurons were transiently transfected by either AR full length (pAR-2000) or deletion reporter plasmids. After 24 h, the cells were treated by either CTL or 10 μM BBR for 24 h, and BBR-induced relative AR reporter activities (% control) were calculated, *n* = 4. *P < 0.05, vs. pAR-2000 group. **(D)** The human ACS-5003 neurons were treated as mentioned by **(B)**, and then cells were harvested for ChIP analysis, *n* = 4. *P < 0.05, vs. CTL group. Data were expressed as mean ± SEM.

We investigated the potential molecular mechanism for DHT-induced ERβ suppression. ERβ promoter deletion constructs were generated and transfected into conditional immortalized human neurons for ERβ reporter activity assay in the presence of either control (CTL) or 10 nM DHT for 24 h, and the DHT-induced relative reporter activities (% control) were calculated. We found that DHT-induced ERβ reporter suppression occurred among the −2,000, −1,600, −1,200, −800, −400, −300 deletion constructs according to Ensembl gene ID: ESR2-201 ENST00000267525.10; the suppression was restored in the −200, −100, and −0 deletion constructs, suggesting that DHT-responsive transcriptional element is located in the range of −300 to 0 on the ERβ promoter (see Figure 2A). We then measured the possible epigenetic changes in the area of −300 to 0 on ERβ promoter. The ChIP analysis showed that there was no significant difference in either histone H3 (K9, K14, K18, K23, K27) or H4 (K5, K8, K12, K16) acetylation (see Supplementary Figure S2A). Also, DHT does not affect histone H4 methylation (see Supplementary Figure S2B). Finally, DHT treatment showed no effect on the methylation of H3K9me3 and H3K27me2, while it increased H3K9me2 and H3K27me3 to 189% and 235%, respectively, compared to the CTL group; both DHT/shAR and DHT/BBR group completely restored this effect (see Figure 2B). This indicates that DHT-mediated ERβ suppression is due to DHT-induced histone methylation on the ERβ promoter through AR.

### BBR-Mediated AR Suppression Is Due to BBR-Induced Histone Methylation on the AR Promoter

We investigated the possible mechanism for BBR-induced AR suppression. AR promoter deletion constructs were generated and transfected into conditional immortalized human neurons for AR BBR reporter activity assay in the presence of either control (CTL) or 10 μM BBR for 24 h, and the BBR-induced relative reporter activities (% control) were calculated. We found that BBR-induced AR reporter suppression occurred among the −2,000, −1,600, −1,200, −800, −400, −300 deletion constructs according to Ensembl gene ID: ARLNC1-205 ENST00000650661.1; the suppression was restored in the −200, −100, and −0 deletion reporter constructs, suggesting that BBR-responsive transcriptional element is located in the area of −300 to 0 on the AR promoter (see Figure 2C). We then measured epigenetic changes in this area. Our results showed that BBR did not affect either histone H3 or H4 acetylation (see Supplementary Figure S3A). as well as histone H4 methylation (see Supplementary Figure S3B). We finally measured the effect of BBR on histone H3 methylation (see Figure 2D). We found that both DHT and DHT/shAR groups had no effect, and BBR treatment had no effect on the methylation of H3K9me2, H3K9me3, and H3K27me3, while it increased H3K27me2 to 219% compared to CTL group. We conclude that BBR-mediated AR suppression is due to BBR-induced histone H3K27me2 methylation on the AR promoter.

### Prenatal Treatment of Either shAR or BBR Ameliorates Prenatal DHT Exposure-Induced Oxidative Stress and Mitochondrial Dysfunction by AR Suppression

**Figure 3:**
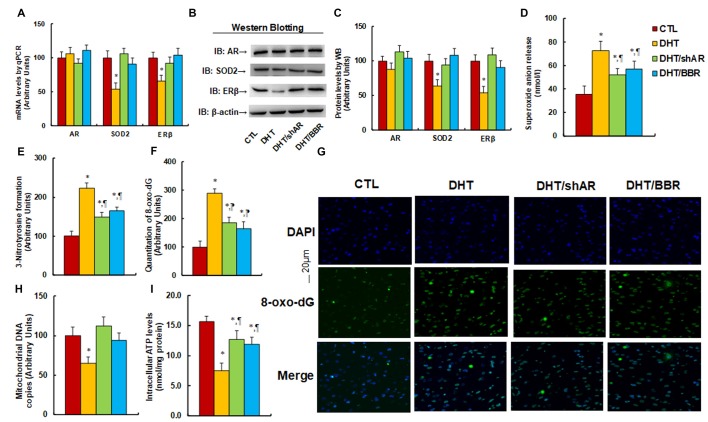
Prenatal treatment of either shAR or BBR ameliorates prenatal DHT exposure-induced oxidative stress and mitochondrial dysfunction by AR suppression. The 2 month-old female rats received either CTL or DHT treatment, which involved either DHT with shAR lentivirus infusion in the amygdala (DHT/shAR) or DHT with BBR injection (DHT/BBR) during the 21-day pregnancy. The subsequent 2 month-old male offspring were sacrificed, and amygdala tissues and/or amygdala neurons were isolated for further analysis. **(A)** The mRNA levels for gene expression of AR, ERβ and SOD2, *n* = 4. **(B)** The representative pictures for western blotting. **(C)** The quantitation of protein levels, *n* = 5. **(D)** *In vivo* superoxide anion release, *n* = 5. **(E)** Quantitation of 3-nitrotyrosine formation, *n* = 5. **(F,G)** The amygdala neurons were isolated for immunostaining by 8-oxo-dG and DAPI. **(F)** Quantitation of 8-oxo-dG staining, *n* = 5. **(G)** Representative pictures for 8-oxo-dG staining (green) and DAPI staining for nuclei (blue). **(H)** Mitochondrial DNA copies, *n* = 4. **(I)** Intracellular ATP levels, *n* = 5. *P < 0.05, vs. CTL group; ^¶^*P* < 0.05, vs. DHT group. Data were expressed as mean ± SEM.

The 2 month-old female rats received either control (CTL) or DHT treatment, which involved either DHT with shAR lentivirus infusion in the amygdala (DHT/shAR) or DHT with BBR injection (DHT/BBR) during the 21-day pregnancy. The subsequent 2-month old male offspring were sacrificed and the brain tissues were isolated for further analysis. We first evaluated the mRNA expression of AR, SOD2, and ERβ in the amygdala. The results showed that prenatal DHT exposure (DHT) decreased expression of SOD2 and ERβ to 54% and 66%, respectively, compared to the CTL group; none of the prenatal treatments had any effect on AR expression, while both the DHT/shAR and DHT/BBR treatments completely restored the DHT-mediated suppression of SOD2 and ERβ (see Figure 3A). We then measured the protein levels for those genes, and an expression pattern similar to that of the mRNA levels was observed (see Figures 3B,C, Supplementary Figure S1B). We then measured oxidative stress, and found that the DHT group showed increased O_2_^.−^ release (see Figure 3D) and 3-nitrotyrosine (3-NT) formation (see Figure 3E) to 202% and 221%, respectively, compared to the CTL group. Furthermore, both the DHT/shAR and DHT/BBR groups partly restored DHT-mediated oxidative stress. We also measured 8-oxo-dG formation using immunohistochemistry (IHC) techniques as another *in vivo* oxidative stress biomarker (see Figures 3F,G). The results showed that the DHT group increased 8-oxo-dG formation to 289%, while DHT/shAR and DHT/BBR group increased 8-oxo-dG formation to 185% and 164%, respectively, partly restoring the DHT-mediated effect. Finally, we evaluated mitochondrial function. The results showed that DHT decreased mitochondrial DNA copies (see Figure 3H) and intracellular ATP levels (see Figure 3I) to 65% and 48%, respectively, compared to the CTL group, while the DHT/shAR and DHT/BBR groups completely restored DHT-induced suppressed mitochondrial replication and partly restored DHT-induced suppressed intracellular ATP levels. We also measured the potential effect of prenatal treatments on gene expression of the hypothalamus and hippocampus. Our results showed that in the DHT group, AR mRNA in the hypothalamus decreased to 73% (see Supplementary Figure S4A), while AR mRNA in the hippocampus decreased to 71% in the DHT/BBR group (see Supplementary Figure S4B) compared to CTL group. There was no effect on the expression of SOD2 and ERβ. Our results suggest that prenatal BBR treatment ameliorates prenatal DHT exposure-induced oxidative stress and mitochondrial dysfunction by AR suppression.

### Prenatal Treatment of Either shAR or BBR Ameliorates Prenatal DHT Exposure-Induced Autism-Like Behavior (ALB) in Offspring

**Figure 4:**
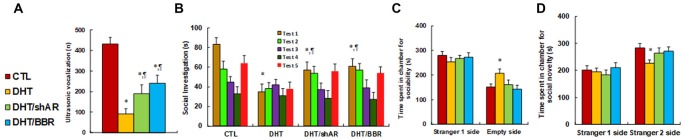
Prenatal treatment of either shAR or BBR ameliorates prenatal DHT exposure-induced autism-like behavior (ALB) in offspring. The 2-month female rats received either CTL or DHT treatment, which involved either DHT with shAR lentivirus infusion in the amygdala (DHT/shAR) or DHT with BBR injection (DHT/BBR) during the 21-day pregnancy and the subsequent 2-month old male offspring were used for ALB analysis. **(A)** Ultrasonic vocalization, *n* = 9. **(B)** Social recognition, seconds spent socially investigating a conspecific [same conspecific in tests 1–4; novel conspecific in test 5 (a new stimulus rat was introduced)], *n* = 9. *P < 0.05, vs. CTL group; ^¶^*P* < 0.05, vs. DHT group. **(C,D)** Three-chambered social tests, *n* = 8. **(C)** Time spent in the chamber for sociability. **(D)** Time spent in the chamber for social novelty. *P < 0.05, vs. CTL group. Data were expressed as mean ± SEM.

We measured the effect of prenatal DHT exposure on ALBs with the prenatal treatment of either shAR or BBR. The results showed that ultrasonic vocalization frequency decreased to 21% in the DHT group compared to the CTL group, while both DHT/shAR and DHT/BBR group partly restored this effect (see Figure 4A). Also, the social recognition showed significant differences for those 4 treatments (*F*_(3,32)_ = 2.741, *P* = 0.041). The *Post hoc* analysis showed that habituation to the same stimulus conspecific (tests 1–4) was significant in CTL group (*F*_(3,32)_ = 4.105, *P* < 0.01), DHT/shAR group (*F*_(3,32)_ = 3.067, *P* = 0.044), and DHT/BBR group (*F*_(3,32)_ = 3.279, *P* = 0.036), but not in DHT group. Dishabituation was significant in CTL group (*F*_(1,8)_ = 4.191, *P* < 0.01), DHT/shAR group (*F*_(1,8)_ = 3.611, *P* = 0.024), and DHT/BBR group (*F*_(1,8)_ = 3.816, *P* = 0.019), but not significant in DHT group (Figure 4B). Also, the three-chambered social tests showed that DHT group increased time spent in the empty side of the chamber for sociability (see Figure 4C) to 137%, and decreased time spent in the Stranger 2 side of the chamber for social novelty (see Figure 4D) to 80%, respectively; while both DHT/shAR and DHT/BBR group completely restored this effect. Additionally, there was no difference in the Stranger 1 side in terms of either sociability or social novelty. The test animal spent most of its time on the Stranger side, with around 300 s for all different treatments (see Figure 4C). A statistically significant difference between the CTL and DHT groups could not be distinguished in evaluating time spent in the Stranger side, while there was a significant difference in time spent on the Empty side, indicating that DHT treatment induces social impairment. Furthermore, we measured the patterns of social behavior in male offspring, finding that DHT treatment decreased time spent Sniffing to 59%, Mounting to 57%, and 60% in total social interaction, with no difference in Grooming partners (see Supplementary Figure S5), and both treatment of the DHT/shAR and DHT/BBR groups completely restored this effect. Our results suggest that prenatal treatment of either shAR or BBR ameliorates prenatal DHT exposure-induced social deficits related to the autistic phenotype in offspring.

### Postnatal BBR Treatment Ameliorates Prenatal DHT Exposure-Induced ALB in Offspring, While Postnatal shAR Treatment Does Not Affect

**Figure 5:**
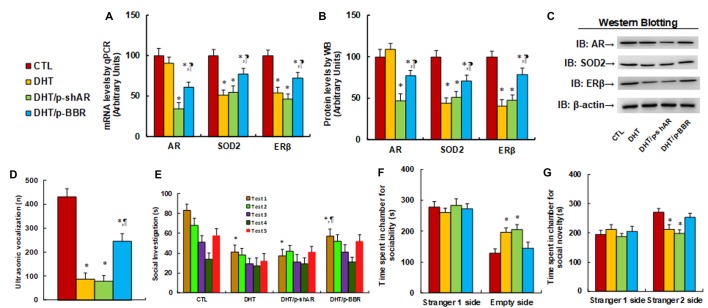
Postnatal BBR treatment ameliorates prenatal DHT exposure-induced ALB in offspring, while postnatal shAR treatment does not affect. The 2-month female rats received either CTL or DHT treatment during their pregnancy, and the subsequent 2-month old male offspring from DHT group received either shAR lentivirus infusion in the amygdala (DHT/p-shAR) or DHT with BBR injection (DHT/p-BBR) for 1 week, and then the male offspring were used for ALB analysis followed by a biomedical analysis of the isolated amygdala. **(A)** The mRNA levels for gene expression of AR, ERβ and SOD2, *n* = 4. **(B)** Quantitation of protein levels, *n* = 5. **(C)** The representative pictures for western blotting for **(B)**. **(D)** Ultrasonic vocalization, *n* = 9. **(E)** Social recognition, seconds spent socially investigating a conspecific [same conspecific in tests 1–4; novel conspecific in test 5 (a new stimulus rat was introduced)], *n* = 9. *P < 0.05, vs. CTL group; ^¶^*P* < 0.05, vs. DHT group. **(F,G)** Three-chambered social tests, *n* = 8. **(F)** Time spent in the chamber for sociability. **(G)** Time spent in the chamber for social novelty. *P < 0.05, vs. CTL group. Data were expressed as mean ± SEM.

We measured the possible effect of postnatal BBR treatment on prenatal DHT exposure-mediated ALB in offspring. The 2-month female rats received either control (CTL) or DHT treatment during their pregnancy, and the subsequent 2-month-old male offspring from the DHT group received either shAR lentivirus infusion in the amygdala (DHT/p-shAR) or DHT with BBR injection (DHT/p-BBR) for 1 week, and then those male offspring were used for ALB analysis followed by a biomedical analysis of the isolated amygdala. We first evaluated the mRNA expression of AR, SOD2, and ERβ in the amygdala. The results showed that prenatal DHT exposure (DHT) decreased expression of SOD2 and ERβ to 51% and 54%, respectively, but had no effect on AR expression. Additionally, postnatal shAR treatment (DHT/p-shAR) decreased AR expression to 34%, indicating a successful gene manipulation by shAR lentivirus knockdown, but did not affect prenatal DHT exposure-mediated suppression of SOD2 and ERβ. On the other hand, postnatal BBR treatment (DHT/p-BBR) decreased AR expression to 61% and partly restored prenatal DHT exposure-mediated suppression of SOD2 and ERβ (see Figure 5A). We then measured the protein levels for those genes, and an expression pattern similar to that of the mRNA levels was observed (see Figures 5B,C, Supplementary Figure S1C). We continued to evaluate the effect of postnatal BBR treatment on ALBs. The ultrasonic vocalization results showed that ultrasonic vocalization frequency decreased to 20% in the DHT group compared to the CTL group, and DHT/p-shAR treatment had no effect, while this effect was partly restored in the DHT/p-BBR group (see Figure 5D). Furthermore, the social recognition tests showed a significant difference between 4 treatments (*F*_(3,32)_ = 2.947, *P* = 0.046), and the *Post hoc* analysis showed that habituation to the same stimulus conspecific (tests 1–4) was significant in CTL group (*F*_(3,32)_ = 4.264, *P* < 0.01) and DHT/p-BBR group (*F*_(3,32)_ = 3.410, *P* = 0.034), but not in DHT and DHT/p-shAR groups. Dishabituation was significant in CTL group (*F*_(1,8)_ = 4.361, *P* < 0.01), and borderline significant in DHT/p-BBR group (*F*_(1,8)_ = 3.017, *P* = 0.037), but was not significant in DHT and DHT/p-shAR groups (see Figure 5E). Also, three-chambered social tests showed that in DHT group, time spent in the empty side of the chamber for sociability (see Figure 5F) increased to 153%, and time spent in the empty side of the chamber for social novelty (see Figure 5G) decreased to 79%, respectively; there was no noticeable effect in DHT/p-shAR group, while this effect was completely restored in DHT/p-BBR group. Also, there was no difference in the Stranger 1 side in terms of either sociability or social novelty. Besides, we measured the repetitive patterns of social behavior in male offspring and found that DHT treatment decreased time spent Sniffing to 57%, Mounting to 45%, and total social interaction to 56%, with no difference in Grooming partners (see Supplementary Figure S6). DHT/p-shAR treatment showed no effect, while DHT/p-BBR treatment partly restored this effect. Our results suggest that postnatal BBR treatment ameliorates prenatal DHT exposure-induced ALB in offspring, but postnatal shAR treatment shows no effect. The ineffectiveness of p-shAR treatment can be explained by the hypothesis that even though the treatment can decrease AR expression in offspring, it cannot restore the epigenetic changes on the ERβ promoter, while p-BBR treatment can potentially penetrate the BBB and modify DHT-induced epigenetic changes.

## Discussion

In this study, the neural progenitor cell ACS-5003 was used to mimic the differentiation of stem cells into neurons during embryo development (Wang et al., 2019). The *in vitro* experiments demonstrate that DHT suppresses ERβ expression through AR-mediated hypermethylation on ERβ promoter, and BBR restores this effect through AR suppression. Furthermore, the *in vivo* experiments show that prenatal DHT exposure induces ALB in offspring, and both prenatal and postnatal BBR treatment ameliorates this effect.

### Prenatal DHT Exposure-Mediated Autism-Like Behavior

We have previously found that male offspring are more sensitive than female offspring in ERβ suppression-mediated ALB (Zou et al., 2017). In this study, we chose male offspring as our experimental target. Here, we report that prenatal androgen exposure may also be a potential risk for ASD development. PCOS is characterized by hyperandrogenism (Kosidou et al., 2017), and many epidemiological studies demonstrated that PCOS is associated with ASD development (Kosidou et al., 2016; Cherskov et al., 2018; Katsigianni et al., 2019). Thus, we hypothesize that prenatal androgen exposure may trigger ASD development. The DHT treatment was used to mimic PCOS conditions during the *in vivo* rat experiments (Mannerås et al., 2007; Noroozzadeh et al., 2017; Paixão et al., 2017). Our results show that prenatal DHT exposure induces ALB in offspring with suppressed expression of ERβ and SOD2 in the amygdala (Zou et al., 2017; Li et al., 2018; Xie et al., 2018). AR knockdown partly diminishes this effect, indicating that DHT induces ALB through the AR signaling pathway. Also, it has been reported that the sex hormone regulates the expression of FOXP1 (Takayama et al., 2014; Fröhlich et al., 2017), SHANK (Berkel et al., 2018), and RORA (Sarachana et al., 2011), which is essential for ASD, further confirming the potential role of the AR signaling pathway on ASD development. This can partly explain why the androgen can be related to male/female bias to ASD (Becker, 2012; Schaafsma and Pfaff, 2014; Mogi et al., 2015). This is the first time that the potential mechanism for PCOS-mediated ASD development has been discovered through AR-mediated ERβ suppression, providing a potential target for PCOS-mediated ASD through AR suppression (Baron-Cohen et al., 2011; Lee et al., 2017; Cherskov et al., 2018; Shay et al., 2018).

### Prenatal-DHT Exposure-Mediated Gene Expression in Autistic Offspring

We have previously found that prenatal progestin exposure suppresses ERβ expression in the amygdala instead of in other brain areas and subsequently contributes to ASD development in offspring, with male offspring being more susceptible than females. Additionally, ERβ overexpression in the amygdala significantly restores prenatal progestin exposure-induced ALB (Zou et al., 2017; Xie et al., 2018), and maternal diabetes-induced autistic offspring show significant SOD2 suppression in the amygdala as well (Wang et al., 2019). Our results indicate that gene expression in the amygdala plays an important role in ASD development. In this study, we supposed that the amygdala should be the most sensitive organ in prenatal DHT exposure-induced autistic offspring; thus, we conducted the related manipulation and analysis of the amygdala in male offspring. Furthermore, our data showed that prenatal DHT treatment affects gene expression of AR, SOD2, and ERβ in the amygdala (see Figure 5A) and also suppresses AR expression in the hypothalamus and hippocampus, but has no effect on ERβ and SOD2 (see Supplementary Figure S4), although the possible reason for this still requires further investigation. Besides, we found that prenatal DHT exposure induces SOD2 suppression in offspring, which is consistent with previous findings that either maternal diabetes (Wang et al., 2019) or prenatal exposure of progestin (Zou et al., 2017; Li et al., 2018) induces SOD2 suppression and oxidative stress in neurons. Treatment of antioxidants such as resveratrol (Xie et al., 2018) partly restores this effect, indicating that oxidative stress is associated with the ASD phenotype, which is consistent with findings from recent literature (Mandic-Maravic et al., 2019; Bjørklund et al., 2020).

### Role of BBR on PCOS-Mediated ASD

BBR is one of the herbal medicine that has been employed for the management of neurodegenerative diseases such as AD and PD due to its multi-faceted defensive capabilities, such as its protective role in atherosclerosis-related to lipid- and glucose-lowering properties (Jiang et al., 2015; Cai et al., 2016). Also, BBR has been reported to inhibit the AR signaling pathway (Li et al., 2011; Tian et al., 2016; Youn et al., 2018), while the related mechanism remains unclear. Besides, BBR has been used for the treatment of PCOS to improve pregnancy outcomes through its effect on insulin resistance (Li et al., 2013; Chen et al., 2017) and normalizing the endocrine and metabolic parameters in PCOS women (An et al., 2014; Arentz et al., 2014; Wang et al., 2016), while there have been no reports on the effect of BBR on PCOS-mediated ASD development. In this study, we report that BBR restores DHT-mediated ERβ suppression by AR suppression and subsequently ameliorates prenatal DHT exposure-mediated ALB in offspring. Both prenatal and postnatal treatment of BBR protects from prenatal DHT exposure-induced ALB in offspring, indicating that BBR may be a potential candidate for the treatment of PCOS-induced ASD. We have previously found that prenatal progestin exposure and maternal diabetes contribute to ASD development through ERβ suppression, and ERβ overexpression in the brain restores this effect, indicating that ERβ suppression may contribute to ASD development (Zou et al., 2017; Li et al., 2018; Xie et al., 2018; Wang et al., 2019). On the other hand, many potential prenatal factors may suppress ERβ expression, and DHT is only one of them. In this case, berberine treatment can only be active for androgen or PCOS-mediated ASD, and may not work efficiently on treating ASD mediated by other factors.

## Conclusion

Prenatal DHT exposure induces ALB in offspring through the AR signaling pathway; both prenatal and postnatal treatment of BBR partly restore this effect by suppression of AR expression. We conclude that BBR ameliorates prenatal DHT exposure-induced ALB by AR suppression. This is the first time that the potential mechanism for PCOS-mediated ASD development has been discovered, and BBR may be a potential clinical treatment for PCOS-mediated ASD patients.

## Data Availability Statement

All datasets generated for this study are included in the article/Supplementary Material.

## Ethics Statement

The animal protocol conformed to US NIH guidelines (Guide for the Care and Use of Laboratory Animals, No. 85-23, revised 1996), and was reviewed and approved by the Institutional Animal Care and Use Committee from Guangzhou University of Chinese Medicine.

## Author Contributions

PY wrote the article. PY, LL, and XL designed, analyzed the data and interpreted the experiments. XL, YL, and ZW performed the vector constructions and gene expression analysis. XC, YL, ZW, and MW performed the statistical analysis and part of the rat experiments. YW, MX, and ML performed gene analysis and part of the mapping analysis. DX, JL, and CW performed the remaining experiments. All authors read and approved the final manuscript.

## Conflict of Interest

The authors declare that the research was conducted in the absence of any commercial or financial relationships that could be construed as a potential conflict of interest.

## Abbreviations

ALB: autism-like behavior
AR: androgen receptor
ASD: autism spectrum disorders
BBR: berberine
ChIP: chromatin immunoprecipitation
DHT: dihydrotestosterone
ERβ: estrogen receptor β
O_2^.-_: superoxide anions
PCOS: polycystic ovary syndrome
ROS: reactive oxygen species
SOD2: superoxide dismutase 2.
