# Supplementary material for: Berberine Ameliorates Prenatal Dihydrotestosterone Exposure-Induced Autism-Like Behavior by Suppression of Androgen Receptor
Source: Front Cell Neurosci. 2020 Apr 9;14:87. doi: 10.3389/fncel.2020.00087 (PMC7161090; doi:10.3389/fncel.2020.00087)
Supplement: Supplementary file 1 [file Table_1.DOCX]

**Berberine Ameliorates Prenatal Dihydrotestosterone Exposure-Induced Autism-Like Behavior by Suppression of Androgen Receptor**

Dongfang Xiang^1,^*, Jianping Lu^2,^*, Chongxia Wei^3,^*, Xiaofan Cai^3^, Yongxia Wang^1^, Yujie Liang^2^, Mingtao Xu^1^, Min Liu^1^, Min Wang^3^, Xuefang Liang^1,#^, Ling Li^3,#^, Paul Yao^2,3,#^

**Supplemental Information**

**Data S1.** METHODS

**Reagents and materials**. The Neural Progenitor Cell (NPC) Origin ATCC-BXS0117 Normal; Human (ATCC# ACS-5003) was obtained from ATCC, and cultured in NPC medium, including 464mL DMEM: F12 (ATCC® [30-2006](http://prod-cms.atcc.org/sitecore/shell/Controls/Rich%20Text%20Editor/Global/Products/E/4/F/D/30-2006.aspx)) supplemented with the Growth Kit for Neural Progenitor Cell Expansion (ATCC® [ACS-3003](https://www.atcc.org/Global/Products/ACS-3003.aspx)) with the following components: 5 mL L-Alanyl-L-Glutamine; 5 mL Non-Essential Amino Acids; 10 mL NPC Growth Kit Component A; 5 mL NPC Growth Kit Component B; 1 mL NPC Growth Kit Component C; 10 mL NPC Growth Kit Component D, together with 100 U/ml penicillin, 100 μg/ml streptomycin and 10% human serum. All cells were maintained in a humidified incubator with 5% CO_2_ at 37°C. In some experiments, the ACS-5003 neurons were conditionally immortalized using a hTERT lentivirus vector with an extended life span to achieve higher transfection efficiency and experimental stability (1, 2).

The antibodies for AR (sc-7305), β-actin (sc-47778), ERβ (sc-137381) and SOD2 (sc-30080) were obtained from Santa Cruz Biotechnology. Antibodies for acetyl-histone H4 K5, K8, K12, and K16 (H4K5,8,12,16ac, #PA5-40084) were obtained from Invitrogen. Antibodies for anti-histone H3 acetyl K9, K14, K18, K23, K27(H3K9,14,18,23,27ac, ab47915), H4K20me1 (ab9051), H4K20me3 (ab9053), H4R3me1 (ab17339), H3K9me2 (ab1220), H3K9me3 (ab8898), H3K27me2 (ab24684) and H3K27me3 (ab6002), H2AX (ab20669) and γH2AX (ab2893) were obtained from Abcam, and 3-nitrotyrosine (3-NT) was measured using the 3-Nitrotyrosine ELISA Kit (ab116691 from Abcam) per manufacturers’ instructions. Protein concentration was measured using the Coomassie Protein Assay Kit (Pierce Biotechnology). Luciferase activity assay was carried out using the Dual-Luciferase™ Assay System (Promega) and the transfection efficiency was normalized using a cotransfected renilla plasmid (3). 5α-dihydrotestosterone (DHT, #D-073-1ML) and Berberine (BBR, B3251) was purchased from Sigma.

**Construction of human AR/ERβ reporter plasmid**. Human genomic DNA was prepared from NPC cells. In order to construct AR/ERβ reporter plasmids, the AR/ERβ gene promoters (2kb upstream of the transcription start site plus first exon) were amplified from Ensembl gene ID: ARLNC1-205 ENST00000650661.1 (for AR) and ESR2-201 ENST00000267525.10 (for ERβ) by PCR and subcloned into the pGL3-basic vector (# E1751, Promega) using restriction sites of Mlu I and Hind III with the following primers: AR forward: 5’-gcgc-acgcgt- cga ggt tag gag ata aag acc -3’ (Mlu I) and AR reverse: 5’- gtac- aagctt- ctt ttc tgt aca tct cta gat -3’ (Hind III); ERβ forward: 5’-gcgc-acgcgt- atttcaagacgagcctggcca -3’ (Mlu I) and ERβ reverse: 5’- gtac- aagctt- ctg ttt aca ggt aag gtg tgt -3’ (Hind III). To map AR or ERβ promoter activity, the related deletion promoter constructs were generated by PCR methods and subcloned into the pGL3-basic vector. All the vectors were verified by sequencing, and detailed information on these plasmids is available upon request (3).

**Preparation of human AR expression lentivirus***.* The cDNA for human AR (obtained from Open Biosystems) was subcloned into the pLVX-Puro vector (from Clontech) with the restriction sites of Xho1 and Xba1 using the below primers: human AR forward primer: 5’- gtac - ctcgag- atg gaa gtg cag tta ggg ctg -3’ (Xho1) and human AR reverse primer: 5’- gtac - tctaga - tca ctg ggt gtg gaa ata gat -3’ (Xba1). The lentivirus for either AR or empty control (CTL) was expressed through Lenti-X™ Lentiviral Expression Systems (from Clontech) per manufacturers’ instructions.

**Preparation of shAR knockdown lentivirus**. The shRNA lentivirus plasmid for human AR (sc-29204-SH), or non-target control (sc-108060) were purchased from Santa Cruz Biotechnology; and the shRNA lentivirus plasmid for rat AR was a kind gift from Dr. Haimou Zhang (from Hubei University). The related lentivirus for shAR or empty control (CTL) were expressed through Lenti-X™ Lentiviral Expression Systems (from Clontech) per manufacturers’ instructions. The purified and condensed lentivirus were used for in vivo gene knockdown. The knockdown efficiency was confirmed by more than 65% of mRNA reduction compared to the control group in rat amygdala cells using real time PCR (see Table S1).

**RT reaction and real-time quantitative PCR.** Total RNA from treated cells was extracted using the RNeasy Micro Kit (Qiagen), and the RNA was reverse transcribed using an Omniscript RT kit (Qiagen). All the primers were designed using Primer 3 Plus software with the Tm at 60°C, primer size of 21bp, and the product length in the range of 140-160bp (see Table S1). The primers were validated with an amplification efficiency in the range of 1.9-2.1, and the amplified products were confirmed with agarose gel. Real-time quantitative PCR was run on iCycler iQ (Bio-Rad) with the Quantitect SYBR green PCR kit (Qiagen). The PCR was performed by denaturing at 95°C for 8 min, followed by 45 cycles of denaturation at 95°C, annealing at 60°C, and extension at 72°C for 10s, respectively. 1 µl of each cDNA was used to measure target genes. β-actin was used as the housekeeping gene for transcript normalization, and the mean values were used to calculate relative transcript levels with the ^ΔΔ^CT method per instructions from Qiagen. In brief, the amplified transcripts were quantified by the comparative threshold cycle method using β-actin as a normalizer. Fold changes in gene mRNA expression were calculated as 2^−ΔΔCT^ with CT = threshold cycle, ΔCT=CT (target gene)-CT(β-actin), and the ΔΔCT =ΔCT (experimental)-ΔCT (reference) (3, 4).

**Western blotting.** Cells were lysed in an ice-cold lysis buffer (0.137M NaCl, 2mM EDTA, 10% glycerol, 1% NP-40, 20mM Tris base, pH 8.0) with protease inhibitor cocktail (Sigma). The proteins were separated in 10% SDS-PAGE and transferred to the PVDF membrane. The membrane was blotted by primary antibodies (1:1000) and then simultaneously incubated with the differentially labeled species-specific secondary antibodies, anti-RABBIT IRDye™ 800CW (green) and anti-MOUSE (or goat) ALEXA680 (red). Membranes were scanned and quantitated by the ODYSSEY Infrared Imaging System (LI-COR, NE) (5).

**Luciferase reporter assay.** 1.0×10^5^ of treated cells were seeded in a 6-well plate with complete medium to grow until they reached 80% confluence. Cells were then cotransfected by 3µg of VEGF full length or deletion reporter constructs, together with 0.2µg of pRL-CMV-Luc *Renilla* plasmid (from Promega). After treatment, the cells were harvested and the luciferase activity assays were carried out using the Dual-Luciferase^TM^ Assay System (Promega), and the transfection efficiencies were normalized using a cotransfected *Renilla* plasmid according to manufacturers’ instructions. The reporter activities for AR and ERβ were calculated (3).

**Chromatin immunoprecipitation (ChIP).** Cells were washed and crosslinked using 1% formaldehyde for 20 min and terminated by 0.1M glycine. Cell lysates were sonicated and centrifuged. 500µg of protein were pre-cleared by BSA/salmon sperm DNA with preimmune IgG and a slurry of Protein A Agarose beads. Immunoprecipitations were performed with the indicated antibodies, BSA/salmon sperm DNA and a 50% slurry of Protein A agarose beads. Input and immunoprecipitates were washed and eluted, then incubated with 0.2mg/ml Proteinase K for 2h at 42˚C, followed by 6h at 65˚C to reverse the formaldehyde crosslinking. DNA fragments were recovered through phenol/chloroform extraction and ethanol precipitation. A ~150bp fragment in the range of -300~0 from the transcription start site on the AR/ERβ promoter was amplified by real-time PCR (qPCR) using the primers provided in Table S1 (3, 4).

**Measurement of ROS generation.** Treated cells were seeded in a 24-well plate and incubated with 10μM CM-H2DCFDA (Invitrogen) for 45 min at 37°C, and then the intracellular formation of reactive oxygen species (ROS) was measured at excitation/emission wavelengths of 485/530nm using a FLx800 microplate fluorescence reader (Bio-Tek). The data was normalized as arbitrary units (3, 6).

**Evaluation of mitochondrial function.**

*Mitochondrial DNA copies.* Genomic DNA was extracted from the amygdala tissue using a QIAamp DNA Mini Kit (Qiagen) and the mitochondrial DNA was extracted using the REPLI-g Mitochondrial DNA Kit (Qiagen). Purified DNA was used for the analysis of genomic β-actin (marker of the nuclear gene) and ATP6 (ATP synthase F0 subunit 6, marker of the mitochondrial gene) respectively using the qPCR method mentioned above. The primers for genomic β-actin: forward 5’-acc aca gct gag agg gaa atc -3’ and reverse 5’- att gcc gat agt gat gac ctg-3’. The primers for ATP6: forward 5’- tag ggc ttc ttc ccc ata cat -3’ and reverse 5’- tta gtg aga tgg ggg ttc ctt-3’. The mitochondrial DNA copies were obtained from relative ATP6 copies that were normalized by β-actin copies using the ^ΔΔ^CT method.

*Intracellular ATP level.* The intracellular ATP level was determined using the luciferin/luciferase-induced bioluminescence system. An ATP standard curve was generated at concentrations of 10^-12^-10^-3^M. Intracellular ATP levels were calculated and expressed as nmol/mg protein (6).

**In vivo rat experiments.** Sprague Dawley rats were obtained from Guangdong Medical Animal Center, and maintained under standard 12h light/dark cycles and given ad libitum access to food and water. The animal protocol conformed to US NIH guidelines (Guide for the Care and Use of Laboratory Animals, No. 85-23, revised 1996), and was reviewed and approved by the Institutional Animal Care and Use Committee from Guangzhou University of Chinese Medicine.

Rat Protocol 1: Prenatal treatment. The 2-month female rats were monitored for estrous cycles with daily vaginal smears. Only rats with at least two regular 4-5 day estrous cycles were included in the studies. Rats were anesthetized with a mixture of ketamine (90 mg/kg) and xylazine (2.7 mg/kg) intraperitoneal injection, and received treatments consisting of 60-day time release pellets (Innovative Research of America) that were implanted subcutaneously via a ~3mm incision on the dorsal aspect of the neck. Hormone pellets contained 5mg of either dihydrotestosterone (DHT, #A-161), or vehicle pellets (CTL) containing the same matrix but with no hormone (7). Furthermore, the rats were implanted with a guide cannula targeting the amygdala (26 gauge; Plastics One) (8). The following coordinates were chosen for the amygdala: −2.0mm posterior to bregma, ±4.2mm from the midline, and −7.2 mm from the skull surface on which it was based. Cannula was attached to the skull with dental acrylic and jeweler’s screws and closed with an obturator (9). An osmotic minipump (Alzet model 2002; flow rate 0.5 μl/h; Cupertino, CA) connected to a 26-gauge internal cannula that extended 1 mm below the guide was implanted and used to deliver AR knockdown (shAR) or empty (EMP) lentivirus. Vehicle consisting of artificial cerebrospinal fluid (aCSF; 140 mM NaCl, 3 mM KCl, 1.2 mM Na2HPO4, 1 mM MgCl2, 0.27 mM NaH2PO4, 1.2 mMCaCl2, and 7.2 mM dextrose, pH 7.4) was used for the infusion of the lentivirus. Infusion (flow rate 0.5 µl/h) begun immediately after placement of the minipump. 0.5μl of total 2×10^3^ cfu of lentivirus was infused for 1 hour. Cannula placement was verified histologically postmortem by the injection of 0.5μl of India ink (volume matched drug delivery in the experiments). Rats whose dye injections were not located in the amygdala were excluded from the data analysis. After 1 week of surgery recovery, the female rats were mated with proven male rats, and the successful pregnancy was confirmed by examining the vaginal plugs, which was designated as day 0 of pregnancy. The dams were then treated with vehicle (DMSO, 1 mL/kg) or BBR (10 mg/kg, dissolved in DMSO) by intraperitoneal (i.p.) injection every 2 days starting from day 1. The experimental rats were separated into 4 groups (10 per group). Group 1: CTL rats with empty control lentivirus infusion (CTL); Group 2: DHT rats with empty control lentivirus infusion (DHT); Group 3: DHT rats with shAR knockdown lentivirus infusion (DHT/shAR); Group 4: DHT rats with BBR injection (DHT/BBR). The amygdala neurons were isolated on embryonic day 18 (E18) as described below. After birth, the male offspring were separated from the dams on day 21 and fed until 7-8 weeks old for further experiments. The offspring were then used for autism-like behavior tests. After that, the offspring were sacrificed, and the different brain tissues, including the amygdala, hypothalamus and hippocampus were isolated, flash frozen in dry ice, and then stored in a −80°C freezer for the analysis of gene expression, immunohistochemistry, superoxide anion release, DNA damage and mitochondrial function (4).

Rat Protocol 2: Postnatal treatment. The 2-month female rats were prenatally exposed to either CTL or DHT during their 21-day pregnancy period as described by Rat Protocol 1. 2-week old male offspring from the above dams were infused with either AR knockdown (shAR) or empty (EMP) lentivirus or treated with vehicle (VEH, 1 mL/kg of DMSO) or BBR (10 mg/kg, dissolved in DMSO) by intraperitoneal (i.p.) injection every 2 days as mentioned in Rat Protocol 1. The experimental rats were separated into 4 groups (10 per group). Group 1: CTL rats with empty control lentivirus infusion (CTL); Group 2: DHT rats with empty control lentivirus infusion (DHT); Group 3: DHT rats with postnatal shAR knockdown lentivirus infusion (DHT/p-shAR); Group 4: DHT rats with postnatal BBR injection (DHT/p-BBR). After 2 weeks of lentivirus and BBR treatment, the offspring were then used for behavior tests and biomedical analysis, as discussed in Rat Protocol 1 (4).

**Animal behavior test.** The animal behavior test of offspring was carried out at 7-8 weeks of age. Autism-like behavior was evaluated as described below.

*Ultrasonic vocalizations (USVs)*. The USVs of neonates were examined during brief maternal separation on postnatal day 7. USVs from individually-isolated pups were recorded using an externally polarized condenser microphone with a frequency range of 30-300kHz that was attached 15-20cm above the floor of an isolation chamber. The microphone was connected to the Avisoft-UltrasoundGate recording software (Avisoft Bioacoustics, Germany) and the pup-emitted calls were recorded to WAV sound files using parameters optimized for rats. Pups were individually placed in the sound-proof chambers and calls were recorded for 300s. Data transformation on the number of USVs were analyzed using a generalized linear model with a negative binomial distribution and a log-link function (10, 11).

*Social recognition*. Social recognition is defined by reduced time spent investigating a familiar conspecific as a result of social habituation, and subsequent reinstatement of investigation when a novel intruder is introduced (dishabituation). Unfamiliar age- and sex-matched intact stimulus rats were placed in wire mesh containers. Before the test, the stimulus rats were gently habituated to being in the container and focal rats were habituated to having an empty container in their home cage. Each focal rat was tested five times (tests 1-5) in their home cage, in which a container with a stimulus rat was introduced. Each test lasted 5 min and the tests were repeated with a 15-min interval. During the 15-min interval the same empty container was placed back in the home cage of the focal rat. In the first four tests the same stimulus rat was used, whereas for the fifth test, the stimulus rat was replaced with another unfamiliar sex- and age-matched conspecific. The placement of the containers over the five tests was kept constant. During the tests the rats were left undisturbed and their behavior was videotaped and subsequently scored using JWatcher software program. Social investigation was defined as sniffing the wire mesh part of the container (11, 12).

*Three-chambered social test*. 7-8 week old rats were used to assess sociability and the preference for social novelty. Target subjects (Stranger 1 and Stranger 2) were 7-8 week old rats habituated to being placed inside wire cages for 3 days prior to beginning of testing. Test rats were habituated to the testing room for at least 45 min prior to the start of behavioral tasks. For the sociability test, the test animal was introduced to the middle chamber and left to habituate for 5 min, after which an unfamiliar rat (Stranger 1) was introduced into a wire cage in one of the side-chambers and an empty wire cage on the other side-chamber. The test animal was allowed to freely explore all 3 chambers over a 10 min session. Following this, a novel stranger rat (Stranger 2) was introduced into the previously empty wire cage and the test animal was again left to explore for a 10 min session. Parameters scored include time spent in each chamber and number of entries into the chambers. Time spent in each chamber and track maps were calculated using the automated SMART software (12).

*Social interaction (SI) test.* The subjects (Test and Stranger) were separately habituated to the arena for 5 min before the test. During each test, the rats were placed into the apparatus over a period of 20 min and the time spent following, mounting, grooming, and sniffing any body parts of the other rat was taken as an indicator of social engagement, and the social interaction time was calculated and analyzed using EthoVision XT animal tracking software (Noldus, USA) (13). The animal used as the “Stranger” was used only once, and was a Sprague Dawley rat of the same gender, weight, and age, with no previous contact with the test rats (4, 14-16).

**In vitro primary culture of amygdala neurons**. Amygdala tissues were dissected from the rat on embryonic day 18 (E18 rats). Tissues were treated with 0.05% trypsin EDTA for 15 min at 37°C. Trypsin EDTA was replaced with soybean trypsin inhibitor (Sigma) for 5 min at 37°C to stop the reaction. This was then replaced with supplemented Neurobasal A (Invitrogen) followed by mechanical dissociation. Cells were then resuspended in culture media, including Neurobasal A, B27, 1×GlutaMAX and 100 U/ml Pen/Strep (from Invitrogen), and then the cells were incubated at 37°C, 5% CO2 (17). The isolated amygdala neurons were used for immunostaining, the analysis of DNA methylation, epigenetic changes by ChIP assay on the ERβ promoter (4).

**Immunostaining**. The isolated amygdala neurons were transferred to cover slips for incubation under growth conditions, the neurons were washed with PBS, fixed in 4% paraformaldehyde for 20 min, and incubated with 0.3% Triton X-100 in PBS for 15 min. After blocking with normal goat serum, sections with 8-oxo-dG anti-mouse antibody (1:100, # 4354-MC-050, from Novus Biologicals) were incubated for 12h at 4°C and subsequently with secondary antibody Alexa Fluor 488. The cover slips were then mounted by antifade Mountant with DAPI (staining nuclei, in blue). The photographs were taken by using a [Confocal Laser Microscope](https://www.sogou.com/link?url=DSOYnZeCC_qw-OVKG_MsR3KENashJ6PPMhOejy_Q5JJflCntg_rzjU2lo9-QKkufX5Qp7YP6841C08P_Gzn4lQD4cR4JDdkk5sef3Ee0PfoOX3hBKf-DUA..) (Leica, 20x lens) and quantitated by Image J. software.

**In vivo superoxide anion (O_2_^.-^) release**. Superoxide anion release from the amygdala tissue was determined by a luminol-EDTA-Fe enhanced chemiluminescence (CL) system supplemented with DMSO-TBAC (Dimethyl sulfoxide-tetrabutyl-ammonium chloride) solution for extraction of released O_2_^.-^ from tissues, as described previously. The superoxide levels were calculated from the standard curve generated by the xanthine/xanthine oxidase reaction (6).

**Statistical analysis**. The data was given as mean ± SEM, and all the experiments were performed at least in quadruplicate unless indicated otherwise. The one-way analysis of variance (ANOVA) followed by the Turkey−Kramer test was used to determine statistical significance of different groups, and the two-way ANOVA followed by the Bonferroni post hoc test was used to determine the effect of social recognition by SPSS 22 software, and a *P* value of <0.05 was considered significant.

REFERENCES

1. Bodnar AG, Ouellette M, Frolkis M, Holt SE, Chiu CP, Morin GB, Harley CB, Shay JW, Lichtsteiner S, and Wright WE. Extension of life-span by introduction of telomerase into normal human cells. *Science.* 1998;279(5349):349-52.

2. Kong D, Zhan Y, Liu Z, Ding T, Li M, Yu H, Zhang L, Li H, Luo A, Zhang D, et al. SIRT1-mediated ERbeta suppression in the endothelium contributes to vascular aging. *Aging Cell.* 2016;15(6):1092-102.

3. Zhang H, Li L, Li M, Huang X, Xie W, Xiang W, and Yao P. Combination of betulinic acid and chidamide inhibits acute myeloid leukemia by suppression of the HIF1alpha pathway and generation of reactive oxygen species. *Oncotarget.* 2017;8(55):94743-58.

4. Zou Y, Lu Q, Zheng D, Chu Z, Liu Z, Chen H, Ruan Q, Ge X, Zhang Z, Wang X, et al. Prenatal levonorgestrel exposure induces autism-like behavior in offspring through ERbeta suppression in the amygdala. *Mol Autism.* 2017;8(46.

5. Ceradini DJ, Yao D, Grogan RH, Callaghan MJ, Edelstein D, Brownlee M, and Gurtner GC. Decreasing intracellular superoxide corrects defective ischemia-induced new vessel formation in diabetic mice. *J Biol Chem.* 2008;283(16):10930-8.

6. Yao D, Shi W, Gou Y, Zhou X, Yee Aw T, Zhou Y, and Liu Z. Fatty acid-mediated intracellular iron translocation: a synergistic mechanism of oxidative injury. *Free Radic Biol Med.* 2005;39(10):1385-98.

7. Moran AL, Nelson SA, Landisch RM, Warren GL, and Lowe DA. Estradiol replacement reverses ovariectomy-induced muscle contractile and myosin dysfunction in mature female mice. *J Appl Physiol.* 2007;102(4):1387-93.

8. Neal-Perry G, Yao D, Shu J, Sun Y, and Etgen AM. Insulin-like growth factor-I regulates LH release by modulation of kisspeptin and NMDA-mediated neurotransmission in young and middle-aged female rats. *Endocrinology.* 2014;155(5):1827-37.

9. Hu M, Richard JE, Maliqueo M, Kokosar M, Fornes R, Benrick A, Jansson T, Ohlsson C, Wu X, Skibicka KP, et al. Maternal testosterone exposure increases anxiety-like behavior and impacts the limbic system in the offspring. *Proc Natl Acad Sci U S A.* 2015;112(46):14348-53.

10. Silverman JL, Yang M, Lord C, and Crawley JN. Behavioural phenotyping assays for mouse models of autism. *Nat Rev Neurosci.* 2010;11(7):490-502.

11. Schaafsma SM, Gagnidze K, Reyes A, Norstedt N, Mansson K, Francis K, and Pfaff DW. Sex-specific gene-environment interactions underlying ASD-like behaviors. *Proc Natl Acad Sci U S A.* 2017;114(6):1383-8.

12. Moy SS, Nadler JJ, Perez A, Barbaro RP, Johns JM, Magnuson TR, Piven J, and Crawley JN. Sociability and preference for social novelty in five inbred strains: an approach to assess autistic-like behavior in mice. *Genes Brain Behav.* 2004;3(5):287-302.

13. Mufford JT, Paetkau MJ, Flood NJ, Regev-Shoshani G, Miller CC, and Church JS. The development of a non-invasive behavioral model of thermal heat stress in laboratory mice (Mus musculus). *J Neurosci Methods.* 2016;268(189-95.

14. Bahi A. Sustained lentiviral-mediated overexpression of microRNA124a in the dentate gyrus exacerbates anxiety- and autism-like behaviors associated with neonatal isolation in rats. *Behav Brain Res.* 2016;311(298-308.

15. Bahi A. Hippocampal BDNF overexpression or microR124a silencing reduces anxiety- and autism-like behaviors in rats. *Behav Brain Res.* 2017;326(281-90.

16. Xie W, Ge X, Li L, Yao A, Wang X, Li M, Gong X, Chu Z, Lu Z, Huang X, et al. Resveratrol ameliorates prenatal progestin exposure-induced autism-like behavior through ERbeta activation. *Mol Autism.* 2018;9(43.

17. Hay CW, Shanley L, Davidson S, Cowie P, Lear M, McGuffin P, Riedel G, McEwan IJ, and MacKenzie A. Functional effects of polymorphisms on glucocorticoid receptor modulation of human anxiogenic substance-P gene promoter activity in primary amygdala neurones. *Psychoneuroendocrinology.* 2014;47(43-55.

**Table S1. Sequences of primers for the real time quantitative PCR (qPCR)**

| Gene | Species | Analysis | Forward primer (5'→3') | Reverse primer (5'→3') |
| --- | --- | --- | --- | --- |
| β-actin | Human | mRNA | gatgcagaaggagatcactgc | atactcctgcttgctgatcca |
| AR | Human | mRNA | acagcagcaggaagcagtatc | accgacactgccttacacaac |
| ERβ | Human | mRNA | atgatgatgtccctgaccaag | acatcagccccatcattaaca |
| SOD2 | Human | mRNA | gcctacgtgaacaacctgaac | tgaggtttgtccagaaaatgc |
| AR | Human | ChIP | tgattttggacttgcccatag | cccatcattttcttgctttga |
| ERβ | Human | ChIP | ctcacattcccactcctctga | gaaacacagaagatattgccaag |
| β-actin | Rat | mRNA | ttccttcctgggtatggaatc | cttctgcatcctgtcagcaat |
| AR | Rat | mRNA | ctacggagctctcacttgtgg | taacatttccggagacgacac |
| ERβ | Rat | mRNA | tcagcatgaagtgcaaaaatg | ggttctgggagctctctttgt |
| SOD2 | Rat | mRNA | caactcaggttgctcttcagc | ctcaaaagacccaaagtcacg |

FIGURE S1

**Figure S1. Representative pictures of full blots for Western Blotting.** (a) Representative full blots for Figure 1c. (b) Representative full blots for Figure 3b. (c). Representative full blots for Figure 5c.

FIGURE S2

**Figure S2. DHT-mediated epigenetic changes on the ERβ promoter.** The human ACS-5003 neurons were treated by control (CTL), DHT (10nM), DHT with AR knockdown by lentivirus (DHT/shAR), CTL with AR overexpression by lentivirus (CTL/↑AR), CTL with 10µM BBR (CTL/BBR), or DHT with BBR (DHT/BBR) for 24 hours, and cells were harvested for ChIP analysis. (a) DHT-mediated histone acetylation on the ERβ promoter using H3K9,14,18,23,27ac and H4K5,8,12,16ac antibodies, n=4. (b) DHT-mediated histone H4 methylation on the ERβ promoter, n=4. Data were expressed as mean ± SEM.

FIGURE S3

**Figure S3. DHT-mediated epigenetic changes on the AR promoter.** The human ACS-5003 neurons were treated by control (CTL), DHT (10nM), DHT with AR knockdown by lentivirus (DHT/shAR), CTL with AR overexpression by lentivirus (CTL/↑AR), CTL with 10µM BBR (CTL/BBR), or DHT with BBR (DHT/BBR) for 24 hours, and cells were harvested for ChIP analysis. (a) DHT-mediated histone acetylation on the AR promoter using H3K9,14,18,23,27ac and H4K5,8,12,16ac antibodies, n=4. (b) DHT-mediated histone H4 methylation on the AR promoter, n=4. Data were expressed as mean ± SEM.

FIGURE S4

**Figure S4. Prenatal DHT treatment does not affect the gene expression of AR, SOD2 and ERβ in the hypothalamus and hippocampus.** The 2 month-old female rats received control (CTL), DHT treatment, DHT with shAR lentivirus infusion in the amygdala (DHT/shAR), or DHT with BBR injection (DHT/BBR) during the 21-day pregnancy, and the subsequent 2-month-old male offspring were sacrificed, and the tissues of hypothalamus and hippocampus were isolated for mRNA analysis. (a) The mRNA levels in hypothalamus, n=4. (b) The mRNA levels in hippocampus, n=4. Data were expressed as mean ± SEM.

FIGURE S5

**Figure S5. Prenatal treatment of either shAR or BBR ameliorates prenatal DHT exposure-induced autism-like behavior in offspring.** The 2-month female rats received either control (CTL) or DHT treatment, which involved either DHT with shAR lentivirus infusion in the amygdala (DHT/shAR) or DHT with BBR injection (DHT/BBR) during the 21-day pregnancy, and the subsequent 2-month old male offspring were used for social interaction (SI) test. The time spent following, mounting, grooming, and sniffing any body parts of the other rat was calculated. *, *P*<0.05, vs CTL group; ¶, *P*<0.05, vs DHT group. Data were expressed as mean ± SEM.

FIGURE S6

**Figure S6.** **Postnatal BBR treatment ameliorates prenatal DHT exposure-induced autism-like behavior in offspring, while postnatal shAR treatment has no effect.** The 2-month female rats received either control (CTL) or DHT treatment during their pregnancy, and the subsequent 2-month old male offspring from DHT group received either shAR lentivirus infusion in the amygdala (DHT/p-shAR) or DHT with BBR injection (DHT/p-BBR) for 1 week, and then the male offspring were used for social interaction (SI) test. The time spent following, mounting, grooming, and sniffing any body parts of the other rat was calculated. *, *P*<0.05, vs CTL group; ¶, *P*<0.05, vs DHT group. Data were expressed as mean ± SEM.
